# Association of Variant rs4790904 in Protein Kinase C Alpha with Posttraumatic Stress Disorder in a U.S. Caucasian and African-American Veteran Sample

**DOI:** 10.4172/2167-1044.s4-001

**Published:** 2013-03-15

**Authors:** Yutao Liu, Jacqueline Rimmler, Michelle F. Dennis, Allison E. Ashley-Koch, Michael A. Hauser, Mid-Atlantic Mental Illness Research Education and Clinical Center Workgroup, Jean C. Beckham

**Affiliations:** 1 Center for Human Genetics, Department of Medicine, Duke University Medical Center, Durham, NC, USA; 2 Mid-Atlantic Mental Illness Research, Education and Clinical Center (MIRECC), USA; 3 Durham VA Medical Center, Durham, NC, USA; 4 Department of Psychiatry and Behavioral Sciences, Duke University Medical Center, Durham, NC, USA

**Keywords:** Posttraumatic stress disorder, single nucleotide polymorphism (SNP), Variant rs4790904

## Abstract

**Backgound:**

Posttraumatic stress sisorder (PTSD) is a complex anxiety disorder that can develop after traumatic event exposure. Genetic factors have been associated with PTSD risk. Recently a variant rs4790904 in the protein kinase C alpha (PRKCA) gene has been shown to be associated with PTSD risk. The objective of this study was to replicate this association in a sample of U.S. Afghanistan/Iraq era veterans.

**Methods:**

The genotypes of rs4790904 were evaluated in all trauma-exposed veterans. The sample of U.S. veterans included 428 Caucasians and 533 African-Americans. The statistical analysis was conducted independently in the Caucasian and African-American subjects to evaluate the association with PTSD symptom clusters of B symptoms (re-experiencing), C symptoms (avoidance and numbing), D symptoms (hyperarousal), and with current PTSD diagnosis.

**Results:**

The sample was comprised of 428 Caucasians (186 with current PTSD diagnosis, 242 trauma-exposed controls; median age, 35 years; 15% female) and 533 African-Americans (205 with current PTSD diagnosis, 328 trauma-exposed controls; median age, 41 years; 31% female). We observed a significant correlation between rs4790904 and all three PTSD symptom clusters in the Caucasian population, but no significant association with current PTSD diagnosis. However, these significant associations were with the G allele, rather than the A allele, that was previously reported by de Quervain. A significant association of this variant with current PTSD diagnosis (p=0.046) was detected in the African-American veterans.

**Conclusion:**

We confirmed the correlation between rs4790904 and all three PTSD symptom clusters in the Caucasian but not the African-American population. A significant association with a current diagnosis of PTSD was found in the African-American veterans.

## Introduction

Posttraumatic stress disorder (PTSD) is a complex anxiety disorder characterized by symptoms of re-experiencing, avoidance and numbing, and hyperarousal. Exposure to a traumatic event is a necessary, but insufficient condition for a PTSD diagnosis. In fact, the majority of individuals who are exposed to a traumatic event do not develop PTSD [1]. PTSD has been considered to have multifactorial etiology with the interaction of genetic and environmental factors [2, 3]. This interaction is also called diathesis-stress model, which is formulated as an interaction between genetic inheritance, epigenetic modification and life events factors [4]. The genetic factors may only predispose the individual to PTSD. Exposure to traumatic events is required to trigger the genetic factor into action and to develop PTSD. The involvement of genetic factors has been demonstrated by family studies, twin studies, and many molecular genetic studies [5–7]. A number of genes have been implicated in PTSD pathogenesis, including protein kinase C alpha (PRKCA) [5–8].

de Quervain et al. [8] reported that a single nucleotide polymorphism (SNP) rs4790904 in the PRKCA gene was significantly associated with memory for negative pictures, increased PTSD re-experiencing and avoidance symptoms, and increased risk of current PTSD diagnosis in genocide survivors from Rwanda. The main purpose of our study is to replicate these findings in a trauma-exposed U.S. veteran sample.

## Materials and Methods

The research was reviewed and approved by the Institutional Review Boards at the Salisbury VA, Hampton VA, Durham VA and Duke University Medical Centers. The dataset included 428 Caucasians (186 current PTSD cases, 242 controls; median age, 35 years; 15% female) and 533 African-Americans (205 current PTSD cases, 328 controls; median age, 41 years; 31% female). All individuals were required to have a history of trauma exposure as measured by the Traumatic Life Events Scale (TLEQ) [9]. The TLEQ is a 22-item questionnaire designed to assess exposure and response to traumatic events. Respondents are asked how many times they have experienced each of 21 different traumatic events (DSM-IV Criterion A1 for PTSD), as well as an additional item providing an opportunity to report any other potentially traumatic events. Those endorsing a particular event are also asked whether it met DSM-IV Criterion A2 for PTSD (i.e., it evoked fear, helplessness or horror). Consistent with previous work [10], individual items/exposures were summarized into categories of trauma type, e.g., childhood physical abuse, accident/disaster. In this sample, there were a wide range of endorsed trauma exposure types, including combat, Illness/sudden death, child sexual abuse and violence, accident/ natural disaster, and adult physical and sexual assault. We excluded any individuals self-reported to be neither African-Americans nor Caucasians to reduce the genetic heterogeneity. Current PTSD cases were diagnosed using the Structured Clinical Interview for DSM-IV Disorders (SCID) administered by trained interviewers [11, 12]. In accordance with the DSM-IV [11], PTSD consists of three symptom clusters. These include re-experiencing symptoms (B symptoms), avoidance and numbing symptoms (C symptoms) and hyperarousal symptoms (C symptoms). Total PTSD symptoms and symptom clusters (B, C, or D) were measured using the Davidson Trauma Scale for all veterans including individuals with current PTSD diagnosis and controls [13].

Genomic DNA was extracted from peripheral blood samples via alcohol and salt precipitation using Gentra Systems PUREGENE DNA Purification kit (Qiagen, Valencia, VA, USA). DNA genotypes for rs4790904 were evaluated for all US veterans. Analysis of Hardy- Weinberg equilibrium (HWE) was performed separately for individuals with current PTSD diagnosis and controls from the Caucasian and African-American populations with GDA software [14]. Deviations from HWE may indicate population stratification and potential problems in genotyping. The statistical analysis was conducted using SAS software 9.02 (SAS Institute, Cary, NC, USA) independently in the Caucasian and African-American subjects. Spearman correlation was performed to test the association of PTSD symptoms and symptom clusters (B, C, D) in both current PTSD cases and controls with SNP rs4790904. Chi-square analysis was performed to analyze the association between the genotypes and the current PTSD diagnosis. In addition, a logistic regression using an additive model was also applied to test the association between the genotypes and the current PTSD diagnosis controlling for age, gender, and total number of different types of traumatic events.

## Results and Discussion

Variant rs4790904 was in Hardy-Weinberg equilibrium in both individuals with current PTSD diagnosis and controls of Caucasian and African-American populations. Similar to the original report [8], the A allele was the major allele in both Caucasian and African-American samples. The frequencies of A allele are 78% for Caucasian controls and 82% for Caucasian individuals with current PTSD diagnosis, 54% for African-American controls and 55% for African-American individuals with current PTSD diagnosis. The A allele frequency in Caucasian and African-American veteran controls is very similar to that in dbSNP (73–79% for Caucasians and 57–59% in African-Americans). SNP rs4790904 was significantly associated with total PTSD symptoms (spearman correlation *p*=0.02), PTSD B symptoms (re-experiencing; *p*=0.045), PTSD C symptoms (avoidance and numbing; *p*=0.016), and PTSD D symptoms (hyperarousal; *p*=0.017) in the Caucasian samples including both current PTSD cases and controls, but not the African-American samples (*p*>0.05) (Table 1). However, these significant associations were with the G allele, rather than the A allele reported in the original study [8], as indicated by the negative Spearman ρ value in (Table 1).

We found a significant association between the categorical genotype of SNP rs4790904 with current PTSD diagnosis in the African- Americans (*p*=0.046, *χ^2^*, *df*=2), but not the Caucasians (*p*=0.37, *χ^2^*, *df*=2) (Table 2). However, this association with current PTSD diagnosis failed to be significant after adjusting for the covariates of age, gender, and total number of different types of traumatic life events (*p*>0.05). In addition, the PTSD symptom analysis failed to suggest differential genetic risks for different clusters of PTSD symptoms.

The differences between our study and the original report could potentially be explained by several factors [8]. First, ethnicity varied across the two studies (i.e., Rwandan African *vs.* Caucasian or Rwandan African *vs.* African-American) [15]. Second, effect of trauma exposure differed across the two studies: in the current report, the actual number of different types of traumatic events was significantly related to the diagnosis of PTSD whereas this was not the case in the de Quervain study [8]. Level of trauma exposure is a well-documented predictor of PTSD diagnosis [16], but it is possible that all of the subjects in the de Quervain study were very heavily traumatized (although the range and mean of trauma event types was unreported), suggesting that genetic effects may be more pronounced in a very heavily traumatized, homogeneous population. Based on the diathesis-stress model [4], heavier trauma exposure means increased stress burden, with which the individuals with similar genetic predisposition will have an increase possibility to develop PTSD than with less trauma. It has been hypothesized that trauma type can affect PTSD symptom level and other variables. For example, veterans who engage in or observe atrocities may experience more guilt and shame [17]. Given that the individuals in this study report a range of trauma types, and differences in trauma exposure measurement across the two studies, it would be difficult to evaluate a particular trauma type as it relates to genetic or behavioral expression in the two studies. Third, the study samples differed in proportions of male individuals. The original Rwandan cohort included 163 males (47%) and 184 females (53%) while our US veteran cohort consisted of mostly males (85% in Caucasians and 69% in African-Americans). The more balanced male/female ratio in the African-American veterans was more similar to the original dataset in Rwanda, which might explain the association between variant rs4790904 and PTSD diagnosis. We are actively enrolling more female veterans into our study to address the potential gender imbalance in our sample. Finally, although our cohort was larger than those in the original report, the relative genetic impact of this SNP might vary in different populations, which may require a larger sample size in our study to detect the genetic association. Given differences in the studies in terms of trauma exposure measurement, minority and gender composition, and population type, it is not possible to evaluate the impact of these variables across the studies. However, the possible impact of each of these variables suggest that careful lifetime trauma exposure measurement, as well as matching or controlling for minority, gender and population type will be important in future studies in this area.

In summary, we observed a significant correlation between rs4790904 and all three PTSD symptom clusters in the Caucasian population, although we observed association with the opposite allele than the de Quervain study. We observed no significant association of SNP rs4790904 with current PTSD diagnosis in the Caucasian veterans. A significant association of this variant with current PTSD diagnosis was observed in our African-American veterans. These results underscore the importance of evaluating genetic risk across multiple trauma populations and standardizing construct measurement (e.g., level of trauma exposure) so that studies can be validly compared.

## Figures and Tables

**Table 1:** Correlation of the A allele of rs4790904 with PTSD-related symptoms in a U.S. Caucasian and African-American veteran sample.

	Caucasian (n=428)			African-American (n=533)		
	Re-experiencing	Avoidance & numbing	Hyperarousal	Re-experiencing	Avoidance & numbing	Hyperarousal
Spearman ρ	−0.10	−0.12	−0.12	−0.05	−0.01	−0.03
Significance *P*	0.045	0.016	0.017	0.232	0.774	0.496

**Table 2:** Association between the genotype of variant rs4790904 in the protein kinase C alpha (PRKCA) gene and risk for PTSD in a U.S. Caucasian and African-American veteran sample.

Genotype	Caucasians		African-Americans	
	Control (%) (n=242)	PTSD (%) (n=186)	Control (%) (n=328)	PTSD (%) (n=205)
GG	10 (5.13%)	9 (4.84%)	62 (18.90%)	48 (23.41%)
AG	83 (34.30%)	52 (27.96%)	177 (53.96%)	88 (42.93%)
AA	149 (61.57%)	125 (67.20%)	89 (27.13%)	69 (33.66%)
*p* value	0.37		0.046	
